# 605 Differences in Management and Decision Making in Grease and Scald Burns

**DOI:** 10.1093/jbcr/irae036.239

**Published:** 2024-04-17

**Authors:** Miles Reese, Jessica Burgess, Hemasree Yeluru, Ricardo Rendel, Jay Collins

**Affiliations:** Eastern Virginia Medical School, Norfolk, VA; Eastern Virginia Medical School, Miami, FL; Eastern Virginia Medical School, Norfolk, VA; Eastern Virginia Medical School, Miami, FL; Eastern Virginia Medical School, Norfolk, VA; Eastern Virginia Medical School, Miami, FL; Eastern Virginia Medical School, Norfolk, VA; Eastern Virginia Medical School, Miami, FL; Eastern Virginia Medical School, Norfolk, VA; Eastern Virginia Medical School, Miami, FL

## Abstract

**Introduction:**

There has been a longstanding belief that grease burns are more severe than scald burns due to the higher boiling point of grease. Grease burn patients may remain hospitalized to monitor progression of the burn due to the assumption that these burns may worsen over time, prompting a change in management. The objective of this study was to determine if there was a difference in the management, treatment and outcomes of scald and grease burns.

**Methods:**

This study was a retrospective chart review of patients with thermal burns treated at a level I trauma center. The following data was collected: age, gender, grease or scald burn, total body surface area (TBSA%), depth of burn, evolution of burn depth, type of operative intervention, whether the decision to excise or graft changed from presentation and length of hospital stay (LOS). Data was analyzed using descriptive statistics; means between the two groups were compared using student’s T-Test and categorical variables were compared with chi squared analysis.

**Results:**

A total of 165 patients treated between 2020 and 2023 were included. Of these, 44.8% were scald burns, and 55.2% were grease burns. Patients with scald burns had an increased LOS when compared to those with grease burns (2.96 vs 1.13 d; p = 0.01). However, there was no statistical difference in the rate of change of management for scald burns versus grease burns (6.8% v 3.3%, p=.3). Additionally, patients with scald burns had a slightly increased average TBSA% when compared to grease burns (4.32 vs 3.06; p>0.05). Amongst patients with scald burns, the TBSA% did not have a correlation to the hospital length of stay, however in patients with grease burns, a higher TBSA% was significantly associated with an increased length of hospital stay (p < 0.0001). Amongst patients with scald burns, 77% received a nonexcisional debridement, 16.2% required excision and 6.8% required grafting. For patients with grease burns, 85.7% received a debridement, 9.9% required excision and 6.6% required a skin graft.

**Conclusions:**

This study aimed to highlight differences in presentation, hospital course, and outcomes for patients with grease and scald burns. There were no significant differences in rate of skin grafting or change in management. Although scald burn patients had an increased average length of hospital stay when compared to patients with grease burns, this could be explained by the wide range (0 to 45 days) in the sample due to concomitant medical and social issues which required prolonged hospitalization. Ultimately, this study suggests that grease burns should be treated in similar fashion to scald burns and are not more likely to progress to require a change in management such as excision or graft.

**Applicability of Research to Practice:**

Grease burns should be treated similarly to scald burns in that their management can be based on the initial presentation with a low rate of burn progression necessitating a change in management.

